# Parasitic antigens alter macrophage polarization during *Schistosoma japonicum* infection in mice

**DOI:** 10.1186/1756-3305-7-122

**Published:** 2014-03-25

**Authors:** Jifeng Zhu, Zhipeng Xu, Xiaojun Chen, Sha Zhou, Weiwei Zhang, Ying Chi, Wei Li, Xian Song, Feng Liu, Chuan Su

**Affiliations:** 1 Department of Pathogen Biology and Immunology, Jiangsu Key Laboratory of Pathogen Biology, Nanjing Medical University, 140 Hanzhong Road, Nanjing, Jiangsu 210029, China; 2 Key Laboratory of Enteric Pathogenic Microbiology, Ministry of Health, Institute of Pathogenic Microbiology, Jiangsu Provincial Center for Disease Prevention and Control, Nanjing, Jiangsu 210009, China

**Keywords:** *Schistosoma japonicum*, Liver fibrosis, Macrophage polarization, Schistosomal antigen

## Abstract

**Background:**

Schistosome eggs are trapped in host liver and elicit severe hepatic granulomatous inflammation, which can lead to periportal fibrosis, portal hypertension, hemorrhage, or even death in the host. It was reported that the macrophage plays an important role in host immune responses to schistosome infection. Nitric oxide (NO) produced by classically activated macrophages (M1 macrophages) is cytotoxic to schistosomula and can prevent hepatic schistosomal fibrosis, while arginase-1 (Arg-1) expressed by alternatively activated macrophages (M2 macrophages) promotes hepatic schistosomal fibrosis. However, the dynamics of macrophage polarization, as well as the possible factors that regulate macrophage polarization, during schistosome infection remain unclear.

**Methods:**

We first analyzed M1 and M2-phenotypic markers of peritoneal macrophages from mice infected with *Schistosoma japonicum* (*S. japonicum*) at indicated time points using flow cytometry (FCM) analysis and real-time PCR. Then we treated peritoneal macrophages from normal mice with schistosome worm antigen (SWA) or schistosome soluble egg antigen (SEA) and determined M1 and M2-phenotypic markers, in order to identify macrophage polarization in responding to schistosomal antigens.

**Results:**

In this study, we showed that macrophages were preferentially differentiated into the M1 subtype during the acute stage of *S. japonicum* infection. However, the level of M1 macrophages decreased and M2 macrophages significantly increased during the chronic stage of infection. Furthermore, we showed that SWA favors the generation of M1 macrophages, whereas SEA preferentially promotes M2-polarized phenotype.

**Conclusion:**

These findings not only reveal the parasite antigen-driven dynamic changes in macrophage polarization, but also suggest that manipulation of macrophage polarization may be of therapeutic benefit in controlling excessive hepatic granulomas and fibrosis in the host with schistosomiasis.

## Background

Schistosomiasis is a tropical disease caused by worms of the genus *Schistosoma*. 200 million people are infected worldwide, leading to the loss of 1.53 million disability-adjusted life years [1]. During schistosome infection, in the first three weeks post-infection, the schistosomula migrating in the host are shown to be the main target of immune attack [2-5]. Since 4–5 weeks post-infection the parasites begin to produce eggs. The eggs trapped in host tissues evoke granulomatous responses, which peak around week 8 post-infection, and result in acute symptoms in patients [2,6]. During the chronic phase of the infection (since 11–13 weeks post-infection), increasing collagen deposits in the host liver and fibrosis develops [2,6].

Studies have identified macrophages, one of the main cellular constituents of granulomas [6,7], as critical regulators of liver fibrosis [8-10]. Macrophages are known to retain considerable plasticity and can respond to environmental signals by changing effector phenotypes [11,12]. Macrophages can be categorized into two subpopulations: classically activated macrophages (M1 macrophages) and alternatively activated macrophages (M2 macrophages). M1 macrophages that develop in response to pro-inflammatory stimuli such as LPS or IFN-γ are characterized by the expression of high levels of CD16/32, TNF-α, IL-12, inducible nitric oxide synthase (iNOS) and chemokines such as CXCL9, CXCL10 and CXCL11. M2 macrophages, can be induced by IL-4 or chitin, are characterized by their high expression of mannose receptor (Mrc1 encoding MR, also known as CD206), arginase-1 (Arg-1), IL-10 and chemokines such as CCL2, CCL17 and CCL22 [11-15].

During schistosome infection, M1 macrophages are thought to be cytotoxic to schistosomula as M1 macrophages produced nitric oxide (NO), which is an effector mechanism previously implicated in the killing of schistosomula [3]. M2 macrophages are important regulators of fibrosis via Arg-1 activation. Arg-1 hydrolyses L-arginine to urea and L-ornithine. L-ornithine can be further metabolized to the amino acid proline, which is required for collagen production and thus promotes the development of fibrosis in the liver during schistosomiasis [9,10,16,17]. Nevertheless, L-arginine can also be oxidized by the M1-related enzyme iNOS to NO, which can restrain hepatic fibrosis [10,16,17]. In addition, M2-related chemokines CCL17 and CCL22 are also thought to contribute to the fibrotic response as mice deficient in these chemokines develop smaller granulomas and less fibrosis [18]. Therefore, it is suggested that interventional control of liver fibrosis by regulating macrophage polarization could be a potential therapeutic strategy for schistosomiasis management.

Up to now, the dynamics of macrophage polarization during schistosome infection have not yet been reported. In addition, the possible role of schistosomal antigens in macrophage polarization remains unclear. The present study investigated the kinetics of macrophage polarization in *S. japonicum* infected mice and evaluated the possible role of schistosomal antigens in macrophage polarization, which might provide clues to better control of fibrosis in schistosomiasis.

## Methods

### Ethics statement

Animal experiments were performed in strict accordance with the Regulations for the Administration of Affairs Concerning Experimental Animals (1988.11.1), and efforts were made to minimize suffering. Ethical approval was obtained from the Institutional Animal Care and Use Committee (IACUC) of Nanjing Medical University for the use of laboratory animals.

### Mice infection

Six-week-old female CD-1 mice were purchased from SLAC Laboratory Animal Co. Ltd. (Shanghai, China). Each mouse was infected percutaneously with 12 *S. japonicum* cercariae (Chinese mainland strain). *Oncomelania hupensis* harboring *S. japonicum* cercariae were purchased from the Parasitic Disease Prevention and Research Institute of Jiangsu Province.

### Peritoneal macrophage purification

Peritoneal macrophages were isolated from CD-1 mice as previously described [19]. Briefly, *S. japonicum* infected or normal control mice were sacrificed at 0, 3, 8, or 13 weeks post-infection. Peritoneal exudate cells (PECs) were isolated by peritoneal lavage using PBS with 1% FBS. PECs were obtained by centrifugation at 500 g for 5 min at 4°C and resuspended in ACK lysis buffer (0.15 M NH_4_Cl, 10 mM KHCO_3_, 0.1 mM Na_2_EDTA, pH 7.2) for erythrocyte lysis. Subsequently, cells were centrifuged at 500 g for 5 min at 4°C and finally resuspended in DMEM medium supplemented with 10% FBS, 1% penicillin–streptomycin. PECs were cultured in 6-well plates (Costar, Cambridge, MA) at a density of 5 × 10^6^/well to allow macrophages to adhere. Macrophages were purified by adherence for 2.5 h, at which time media were replaced to remove non-adherent cell. Adherent macrophages were incubated with 5 mM EDTA/PBS for 10 min at 37°C, and then detached by vigorous pipetting to prepare single-cell suspension for purity analysis. As determined by flow cytometry (FCM) analysis using PE-conjugated antibody against mouse F4/80 and APC-conjugated antibody against mouse CD11b (eBioscience, San Diego, CA), the purity of isolated macrophage was >98%.

### *In vitro* treatment of peritoneal macrophage

Peritoneal macrophages were purified from normal mice as described above. For antigen treatment, macrophages were washed with PBS to remove the non-adherent cells. Then PBS, 100 μg/ml SWA, 10 μg/ml SEA, 1 μg/ml LPS (Sigma, St. Louis, MO) or 1000 U/ml IL-4 (Peprotech Inc., Rocky Hill, NJ) were added directly to each well with fresh medium, respectively. Cells were then incubated for 24 h at 37°C. Macrophages treated with LPS or IL-4 was used as positive controls for M1 and M2 macrophages, respectively [14].

### Flow cytometry

Single-cell suspensions were washed in PBS with 1% FBS and adjusted to 1 × 10^6^ cells per 100 μl PBS with 1% FBS. For purity analysis of macrophage, cells were incubated with PE-conjugated antibody against mouse F4/80 and APC-conjugated antibody against mouse CD11b (eBioscience). For M1 and M2 surface marker analysis, cells were incubated with PE-conjugated antibodies against mouse CD16/32 or CD206 (eBioscience). PE- or APC- conjugated rat IgG2a (eBioscience) served as control antibodies. All antibodies were used at 5 μg/ml. The cells were incubated with the antibodies for 30 min at 4°C and washed with PBS. The samples were fixed with 1% paraformaldehyde/PBS and analyzed by using a BD FACSCalibur Flow Cytometer. Results were analyzed using FlowJo software (Tree star Inc, Ashland, OR).

### Real-time PCR

Macrophage total RNA was isolated using TRIzol (Invitrogen, Carlsbad, CA), converted to cDNA using a Reverse Transcription kit (Fermantas Life Sciences, St. Leon-Rot, Baden-Württemberg, Germany) as the manufacturer’s instructions. Quantitative real-time PCR was conducted using FastStart Universal SYBR Green Master (Rox) reagents (Roche Diagnostics, Indianapolis, IN) and a 7300 Real-time PCR machine (Applied Biosystems, Foster City, CA). The reaction conditions consisted of: stage 1, 50°C for 2 min; stage 2, 95°C for 10 min; stage 3, 40 cycles of 95°C for 15 s and 60°C for 1 min, which were concluded by the melting curve analysis process. Fold changes of gene expression were calculated using the 2^-ΔΔCt^ method. The sequences of the primer pairs used in this analysis are as follows: *Tnf*: forward, 5′-catcttctcaaaattcgagtgacaa-3′, reverse, 5′-tgggagtagacaaggtacaaccc-3′; *Il12a*: forward, 5′-gacagtffaggcaccaggcc-3′, reverse, 5′-cagacatcgctgtcccggcg-3′; *Il10*: forward, 5′-actttaagggttacttgggttgc-3′, reverse, 5′-attttcacaggggagaaatcg-3′; *Cxcl9*: forward, 5′-tctcggacttcactccaacaca-3′, reverse, 5′-actccacactgctggaggaaga-3′; *Cxcl10*: forward, 5′-ccgtcattttctgcctcatcc-3′, reverse, 5′-ccctatggccctcattctca-3′; *Cxcl11*: forward, 5′-gaacaggaaggtcacagccatagc-3′, reverse, 5′-tcaactttgtcgcagccgttactc-3′; *Ccl2*: forward, 5′-aagccagctctctcttcctcca-3′, reverse, 5′-aagccagctctctcttcctcca-3′; *Ccl17*: forward, 5′-agtgctgcctggattacttcaaag-3′, reverse, 5′-ctggacagtcagaaacacgatgg-3′; *Ccl22*: forward, 5′-taacatcatggctaccctgcg-3′, reverse, 5′-tgtcttccacattggcacca-3′; *Nos2*: forward, 5′-gccaccaacaatggcaaca-3′, reverse, 5′- cgtaccggatgagctgtgaatt-3′; *Arg1*: forward, 5′-cagaagaatggaagagtcag-3′, reverse, 5′-cagatatgcagggagtcacc-3′; *Irf5*: forward, 5′-aataccccaccaccttttga-3′, reverse, 5′-aataccccaccaccttttga-3′; *Gapdh*: forward, 5′-ggtgaaggtcggtgtgaacg-3′, reverse, 5′-accatgtagttgaggtcaatgaagg-3′.

### Preparation of SWA and SEA

For SWA preparation, *S. japonicum* adult worms were suspended in cold diethyl ether and homogenized on ice using a homogenizer (VirTis Co., Inc., Gardiner, NY). The homogenate was centrifuged at 2,000 g for 5 min at 4°C to remove lipids. The pellet was freeze-thawed several times in PBS mixed with 1 mM PMSF (Roche Diagnostics) and 2ug/ml Leupeptin (Sigma). The homogenate was centrifuged at 20,000 g for 50 min at 4°C and the supernatant was filtered through 0.22 μm filter (Millipore Corporation). Protein concentration of worm extracts were determined by BCA Protein Assay Kit (Bio-Rad) and the extracts were stored at -80°C before use [20].

To prepare SEA, *S. japonicum* eggs were suspended in PBS containing 1 mM PMSF (Roche Diagnostics) and 2 μg/ml Leupeptin (Sigma) and homogenized on ice using a homogenizer (VirTis Co.). The suspension was freeze-thawed several times and centrifuged at 20,000 g for 50 min at 4°C. The supernatant was filtered through 0.22 μm filter (Millipore Corporation). Protein concentration of egg extracts were determined by BCA Protein Assay Kit (Bio-Rad) and the extracts were stored at -80°C before use [21].

### Statistical analysis

Results are shown as the mean ± standard deviation (SD) for at least 3 repeated independent experiments for each group. Statistical comparisons were determined by using SPSS and Student’s t test for independent samples. Significant differences were as follows: *P < 0.05; **P < 0.005; ***P < 0.001.

## Results

### Changes in percentages of M1 and M2 macrophages during *S. japonicum* infection

**Figure 1:**
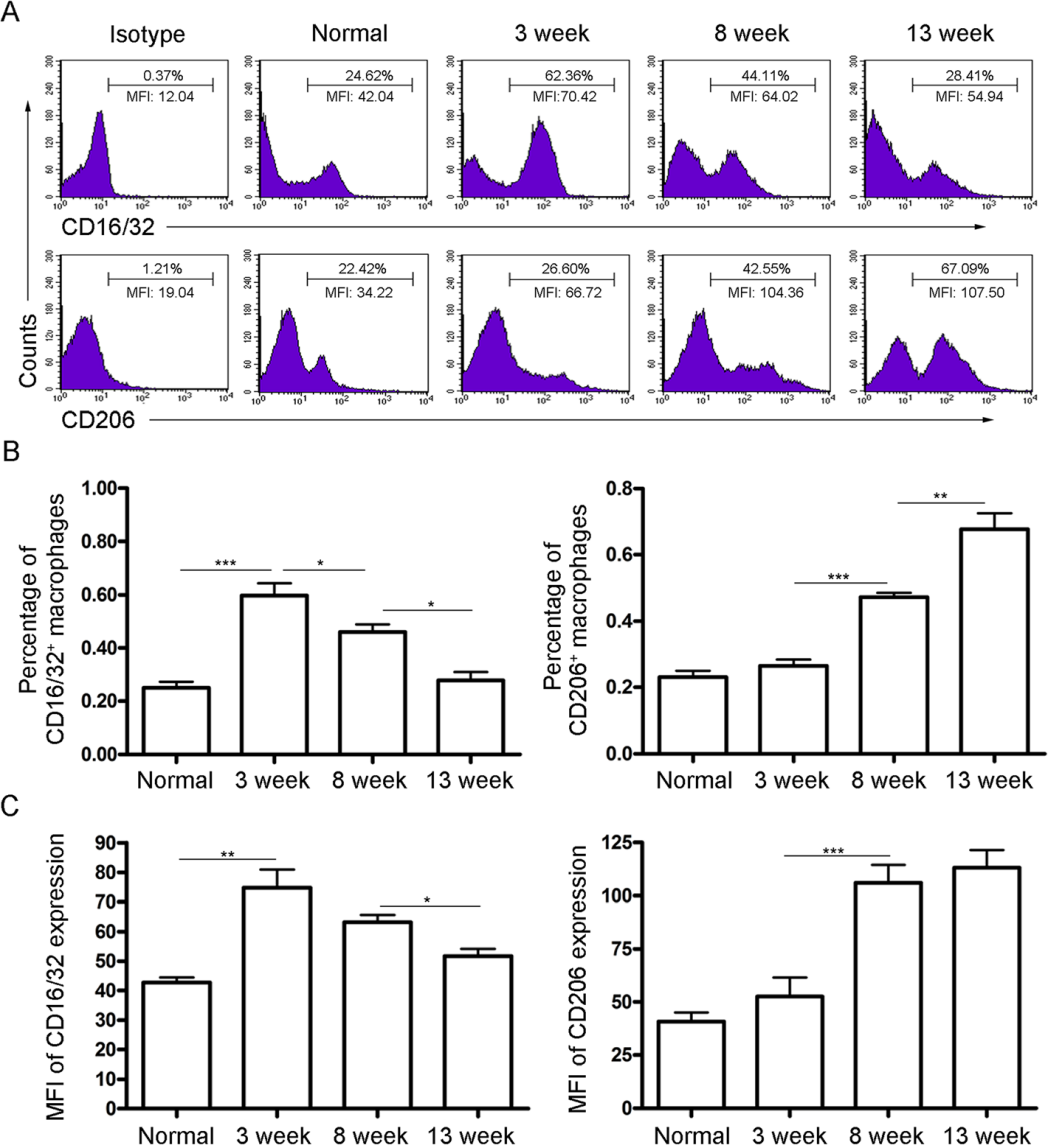
**Changes in percentages of M1 and M2 macrophages during *S. japonicum *infection.** Peritoneal macrophages were purified from normal or *S. japonicum* infected mice at indicated time points post-infection. Expression of CD16/32 (M1) and CD206 (M2) were evaluated by FCM analysis. **(A)** Representative histograms obtained by FCM analysis. **(B)** Percentages of CD16/32^+^ macrophages and CD206^+^ macrophages. **(C)** Mean fluorescence intensity (MFI) of CD16/32 and CD206 expression on macrophages. Data are expressed as the mean ± SD of three independent experiments with 6 mice per group in each experiment.

As shown in Figure 1A and 1B, the percentage of M1 macrophages significantly increased 3 weeks after infection but decreased quickly by 8 weeks post-infection. However, the increase in the percentage of M2 macrophages started at 8 weeks after infection. Similar results were obtained in mean fluorescence intensity (MFI) of CD16/32 and CD206 expression, which reflects the average level of CD16/32 and CD206 expressed on a single M1 or M2 macrophage cell, respectively (Figure 1A and 1C). These results suggest that macrophages are typically skewed from the M1 phenotype at the acute stage of *S. japonicum* infection toward the M2 phenotype at the chronic stage.

### Dynamics of M1 and M2-related chemokines in macrophages during *S. japonicum* infection

**Figure 2:**
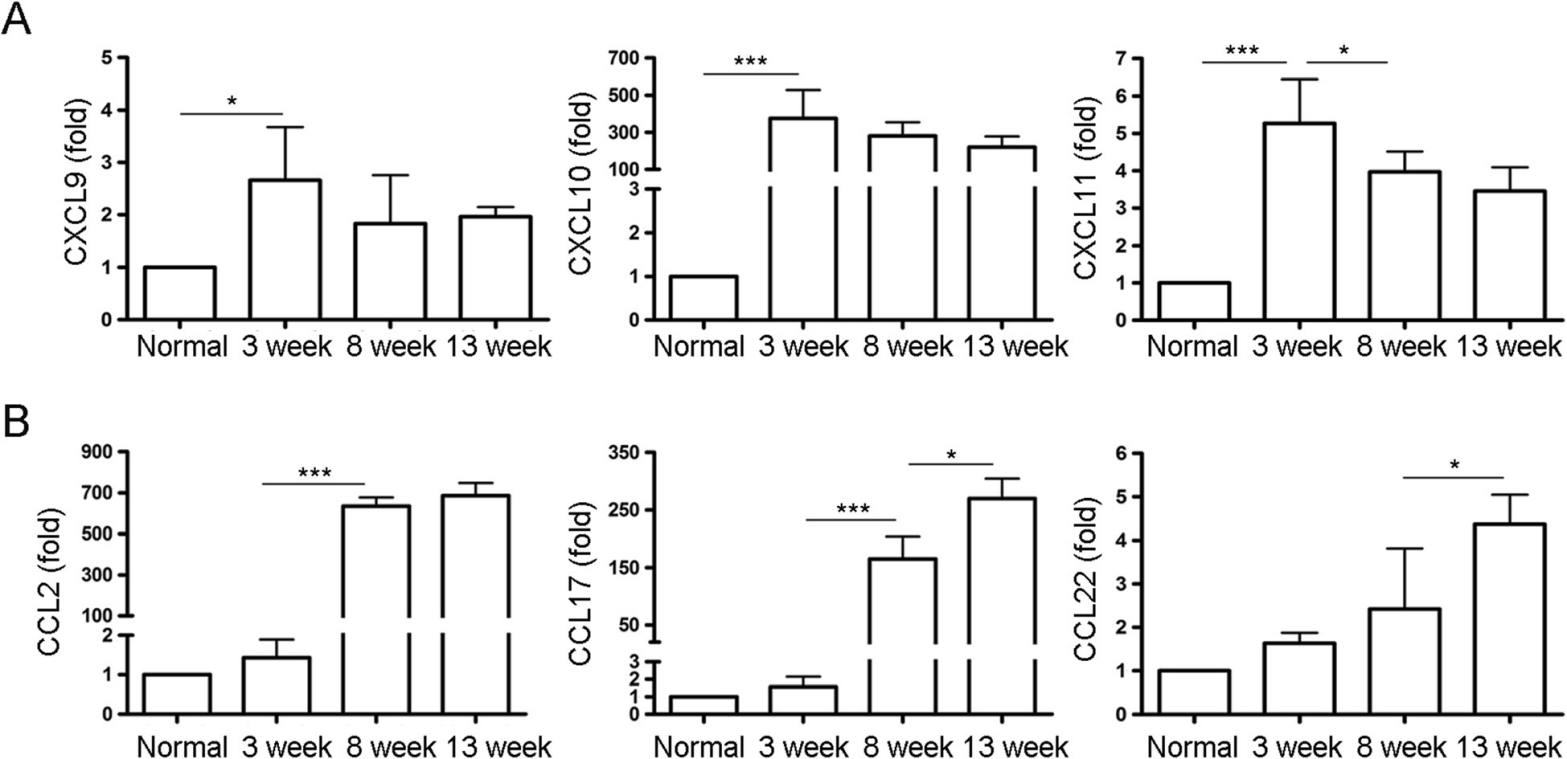
**Dynamics of M1 and M2-related chemokines in macrophages during *S. japonicum *infection.** Peritoneal macrophages were purified from normal or *S. japonicum* infected mice at indicated time points post-infection. Expression of CXCL9, CXCL10, CXCL11 **(A)**, CCL2, CCL17, CCL22 **(B)** mRNA was evaluated by real-time PCR. Transcript levels for each chemokine in macrophages are expressed as fold change over transcript levels in macrophage from normal mice (0 week). Data are expressed as the mean ± SD of three independent experiments with 6 mice per group in each experiment.

The kinetics of M1 and M2 chemokine mRNA expression in peritoneal macrophages of *S. japonicum* infected mice were assessed by real-time PCR. As shown in Figure 2, M1-related chemokines CXCL9, CXCL10, and CXCL11 significantly increased after 3 weeks of infection and then declined. However, M2-related chemokines CCL2, CCL17, and CCL22 progressively increased during the period of 13 weeks infection.

### Dynamics of M1 and M2-related cytokine expression in macrophages during *S. japonicum* infection

**Figure 3:**
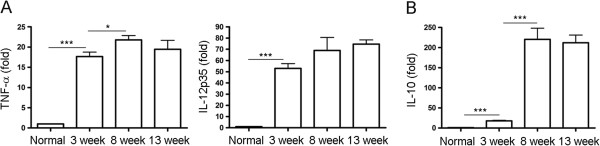
**Dynamics of M1 and M2-related cytokine expression in macrophages during *S. japonicum *infection.** Peritoneal macrophages were purified from normal or *S. japonicum* infected mice at indicated time points post-infection. Expression of TNF-α, IL-12p35 **(A)**, IL-10 **(B)** mRNA was evaluated by real-time PCR. Transcript levels for each cytokine in macrophages are expressed as fold change over transcript levels in macrophage from normal mice (0 week). Data are expressed as the mean ± SD of three independent experiments with 6 mice per group in each experiment.

M1 and M2 cytokine mRNA expressed by peritoneal macrophages of *S. japonicum* infected mice was detected by real-time PCR. Result in Figure 3A showed that 3 weeks after infection, TNF-α and IL-12mRNAs were significantly increased. A further, but slight increase in the expression of both cytokines was observed 8 weeks after infection and the levels of both cytokines were not changed much at 13 weeks. However, a remarkable increase in the expression of IL-10 was observed 8 weeks after infection and maintained at a high level at 13 weeks (Figure 3B).

### Dynamics of M1 and M2-specific enzyme expression in macrophages during *S. japonicum* infection

**Figure 4:**
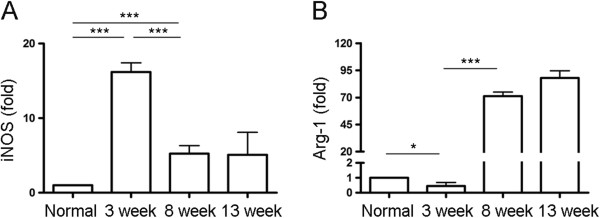
**Dynamics of M1 and M2-specific enzyme expression in macrophages during *S. japonicum *infection.** Peritoneal macrophages were purified from normal or *S. japonicum* infected mice at indicated time points. Expression of iNOS **(A)** and Arg-1 **(B)** mRNA was evaluated by real-time PCR. Transcript levels for each enzyme in macrophages are expressed as fold change over transcript levels in macrophage from normal mice (0 week). Data are expressed as the mean ± SD of three independent experiments with 6 mice per group in each experiment.

As shown in Figure 4A, a significant increase of iNOS, an M1-specific enzyme in macrophages, was observed after 3 weeks of infection, while it was significantly decreased by 8 weeks after infection. In contrast, Arg-1, an M2-specific enzyme in macrophages, showed a slight decrease at week 3 after infection, followed by a marked upregulation at week 8 after infection (Figure 4B).

### Differential effects of SWA and SEA on macrophage polarization

**Figure 5:**
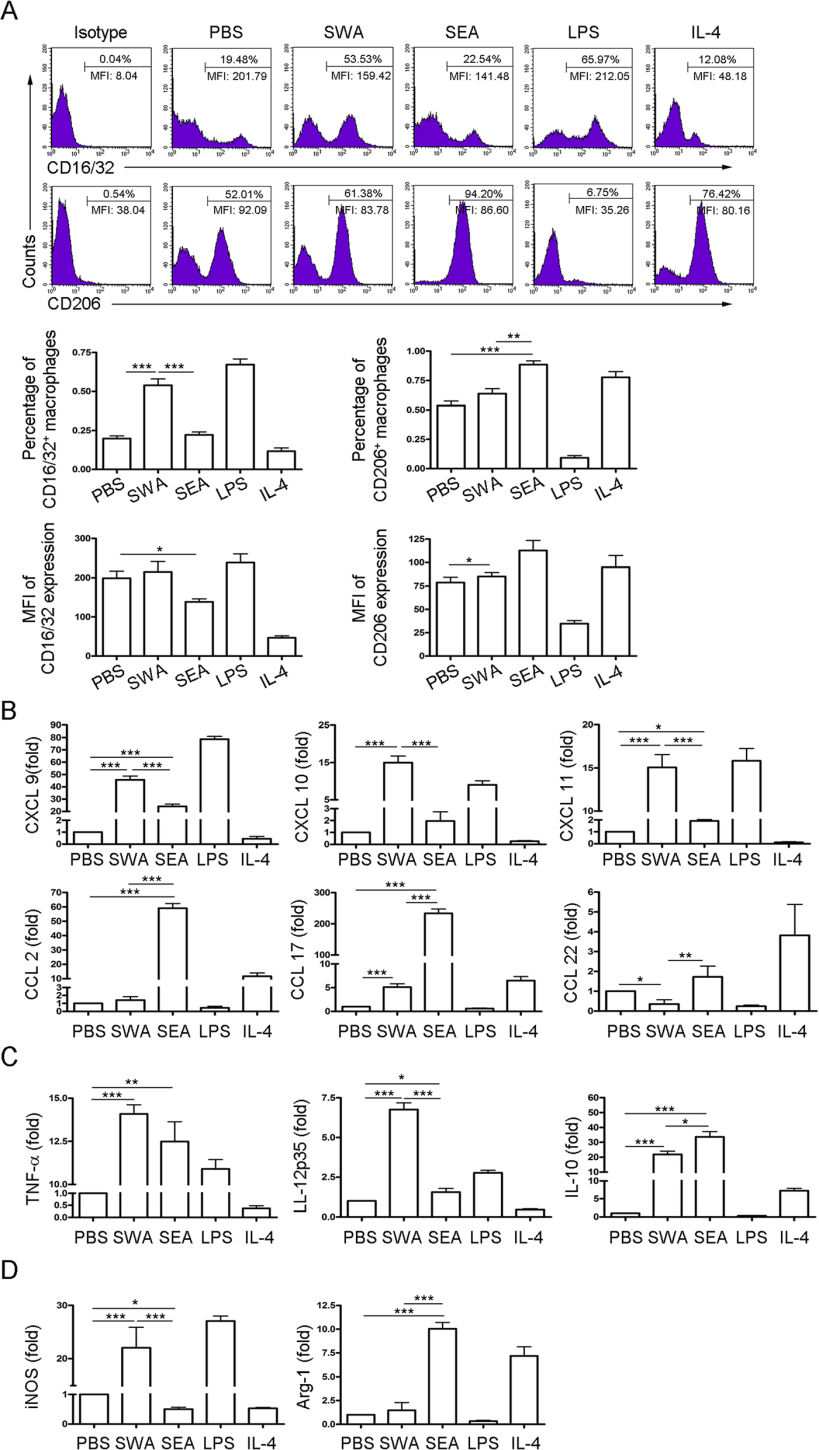
**Differential effects of SWA and SEA on macrophage polarization.** Peritoneal macrophages from normal mice were purified and treated as described in Methods. LPS was included as positive controls for classical activation and IL-4 for alternative activation. The expression of CD16/32 and CD206 were evaluated by FCM analysis **(A)**. Representative histograms obtained by FCM analysis (top row). Percentages of CD16/32^+^ macrophages and CD206^+^ macrophages (middle row). Mean fluorescence intensity (MFI) of CD16/32 and CD206 expression on macrophages (bottom row). The transcript levels for chemokines **(B)**, cytokines **(C)** and enzymes of arginine metabolism **(D)** were evaluated by real-time PCR. The expression levels for each molecule were normalized to the level in macrophages treated with PBS. Data were mean ± SD of three independent experiments.

To investigate whether M1 and M2 macrophages were induced by antigens derived from different stages of the schistosomal life cycle, peritoneal macrophages from normal mice were purified and treated with SWA or SEA *in vitro*. Result showed that compared to PBS control, SWA stimulation significantly increased the percentage of M1 but not M2 macrophages. However, compared to PBS control, SEA stimulation significantly increased the percentage of M2 but not M1 macrophages (Figure 5A). Results in Figure 5B, 5C and 5D showed that SWA stimulation preferentially enhanced the expression of the M1-related chemokines (CXCL9, CXCL10 and CXCL11), cytokines (TNF-α and IL-12), and key enzyme of arginine metabolism (iNOS) in macrophages. In contrast, SEA stimulation preferentially enhanced the expression of the M2-related chemokines (CCL2, CCL17 and CCL22), cytokine (IL-10), and key enzyme of arginine metabolism (Arg-1) in macrophages. Together, these data suggest that during schistosome infection, worm antigens may preferentially elicit pro-inflammatory M1 profile of macrophages, while egg antigens may preferentially induce anti-inflammatory M2 phenotype and downregulate the M1 phenotype.

## Discussion

Schistosomiasis continues to be a significant cause of parasitic morbidity and mortality worldwide [1]. Schistosome egg-induced hepatic granuloma formation can lead to tissue destruction and fibrosis, which causes much of the morbidity and mortality associated with this disease. Macrophages are critical for granuloma formation and the development of hepatic fibrosis during schistosomiasis [2,6]. In this study, we showed that in response to schistosomal antigens, macrophages were typically skewed from M1 towards the M2 polarization during *S. japonicum* infection.

Macrophages are functionally polarized into M1 and M2 phenotypes in response to microorganism infection and host mediators [11-15]. Studies suggest that M1 macrophages are favorable to kill schistosomula by producing NO [3], which also plays a role in preventing hepatic fibrosis [16]. In contrast, M2 macrophages are thought to contribute to schistosome-induced fibrosis via the metabolism of l-arginine to proline and polyamine by Arg-1 [9,10,16,17]. However, factors such as Jmjd3-IRF4 axis involved in the mechanism of M2 polarization have been shown to inhibit M1 polarization and the production of NO by M1 macrophages [12]. Therefore, M2 macrophages may promote the survival of parasites. Considering the peritoneal cavity is suggested by studies as a secondary site of schistosome-induced inflammation [22,23], and the phenotype analysis of peritoneal macrophages is technically easier and widely used in studies of the responses of macrophages related to hepatic injuries such as schistosomiasis, cirrhosis and LPS-induced hepatitis [22,24,25], we analyzed the dynamics of peritoneal macrophage polarization during schistosome infection. Our results showed that a predominant M1 phenotype in peritoneal macrophages was observed in the first 3 weeks post-infection, a period when schistosomula migrate from the skin to the hepatic portal system [2]. These immature worms in migration are reported to be susceptible to activated macrophage-mediated cytotoxicity [3,4,26]. In addition, our results showed that a pronounced M2 activation profile was displayed in peritoneal macrophages at 8 weeks post-infection, a time-point when the severity of hepatic granulomatous response reaches its peak and the fibrosis starts to develop quickly [2,6].

Although we have characterized the dynamics of peritoneal macrophage polarization during schistosome infection, the possible role of schistosomal antigens in macrophage polarization is still unclear. SWA and SEA are two major schistosome antigen mixtures [27-29]. Our results demonstrated that SWA or SEA preferentially induced the macrophages to differentiate to M1- or M2-type cells, respectively. Consistently, recently work demonstrated that hemozoin, a substance isolated from *S. mansoni*, prevented IL-4-inducted alternative activation of bone marrow-derived macrophages in an *in vitro* induction system [30]. M1 macrophages are considered to be pro-inflammatory, whereas M2 macrophages are anti-inflammatory and limit the tissue-damaging activity of M1 macrophages [13,31,32]. Thus, when the host immunity including M1 response cannot eradicate persistent schistosome infection but has to limit collateral severe tissue damage, the switch of macrophage function from killing to repair, which occurs in response to schistosomal antigens, is likely beneficial to the host. Indeed, some other pathogens, such as *Helicobacter pylori*[33,34] and *Trypanosoma cruzi*[35,36], have been shown to induce M2 macrophages to escape killing by M1 macrophages. Therefore, our results also suggested modulation of macrophage polarization by schistosomal antigens as a potential mechanism for both the immune escape of the parasite and the survival strategy of the host during the schistosome infection. Although we have demonstrated the direct role of SWA and SEA in macrophage polarization, previous study also suggest that levels of Th1 and Th2 cytokines, which are affected by SWA and SEA [37-39], are able to modulate M1/M2 skewing [12,40]. Thus, we currently still cannot rule out the possibility that the shift of the M1/M2 polarization may partially through the indirect effect of the Th1/Th2 shift during *S. japonicum* infection.

## Conclusions

In summary, our study described the dynamic changes of macrophage polarization, from M1 to M2 subtypes, during *S. japonicum* infection and revealed distinct roles for schistosomal antigens in regulating macrophage polarization. Our findings not only suggest that signals (antigens) from specific stage of parasite life cycle can skew phenotypic and functional differentiation of macrophages which benefits the parasite and host, but also suggest that these antigens may be potential therapeutic targets for the control of the schistosomal fibrosis.

## Abbreviations

NO: Nitric oxide; Arg-1: Arginase-1; S. japonicum: *Schistosoma japonicum*; SWA: Schistosome worm antigen; SEA: Soluble egg antigen; iNOS: Inducible nitric oxide synthase; PECs: Peritoneal exudate cells; MFI: Mean fluorescence intensity; FCM: Flow cytometry.

## Competing interests

The authors declare that they have no competing interests.

## Authors’ contributions

CS conceived and designed the experiments. JZ and ZX analyzed the data. ZX, XC, SZ, WZ, YC, WL, XS, FL performed the experiments. Manuscript was written by CS and JZ. All authors read and approved the final manuscript.
